# Epidemiological trends in enamel hypomineralisation and molar-incisor hypomineralisation: a systematic review and meta-analysis

**DOI:** 10.1007/s00784-026-06801-2

**Published:** 2026-03-10

**Authors:** Nour Ammar, Karl-Ferdinand Fresen, Falk Schwendicke, Jan Kühnisch

**Affiliations:** 1 Department of Conservative Dentistry, Periodontology and Digital Dentistry, University Hospital, Ludwig-Maximilians Universität München, Munich, Germany; 2 Department of Pediatric Dentistry and Dental Public Health, Faculty of Dentistry, Alexandria University, Alexandria, Egypt

**Keywords:** Epidemiology, Enamel hypomineraisation, Oral health, Cross-sectional studies, Health surveys

## Abstract

**Objective:**

Recently, the systematic review and meta-analysis “Epidemiological Trends in Enamel Hypomineralisation and Molar-Incisor Hypomineralisation: A Systematic Review and Meta-Analysis” was published in Clinical Oral Investigations. In 2025 a reader brought the omission of an epidemiological study from North America to the attention of the author group.

**Materials and methods:**

After checking the eligibility for inclusion we aimed at updating the meta-analysis. After inclusion of this overlooked study the systematic review and meta-analysis covers 139 studies and data from a total of 199,999 participants.

**Results:**

For North America, the MIH prevalence estimate decreased slightly from 0.239 (95% CI: 0.144–0.334) to 0.223 (95% CI: 0.140–0.306). Notably, the global prevalence estimate for MIH remained unchanged at 0.155 (95% CI: 0.144–0.166).

**Conclusion:**

The addition of the overlooked study influenced slightly the MIH outcome for North America but had no impact on the previously drawn conclusions.

**Clinical relevance:**

Enamel hypomineralisation/ Molar incisor hypomineralisation is prevalent across the globe and needs attention by dental practitioners.

## Introduction

Recently, a reader [1] of the above mentioned systematic review and meta-analysis [2] draw attention to the omission of a key U.S. epidemiological study [3] that meets the review’s inclusion criteria and provides nationally relevant data. This study involved an American cohort and reported MIH prevalence and diagnostic criteria consistent with the methods applied by Ammar et al. [2]. Its inclusion could have strengthened the North American dataset, potentially altering the pooled estimates reported by the mentioned study. The study had a standardized clinical examination protocol, conducted by a single calibrated examiner using both the Modified Developmental Defects of Enamel (DDE) Index and the European Academy of Pediatric Dentistry (EAPD) criteria.

We thank Dr. Tagelsir Ahmed and the editors of Clinical Oral Investigations for bringing an overlooked study to our attention. The study by Tagelsir Ahmed et al. [3] was erroneously excluded during the duplicate removal step of the initial review. We have reviewed the study and deemed it eligible for inclusion. Therefore, we aimed at updating the corresponding data in the meta-analysis.

## Results

**Fig. 1:**
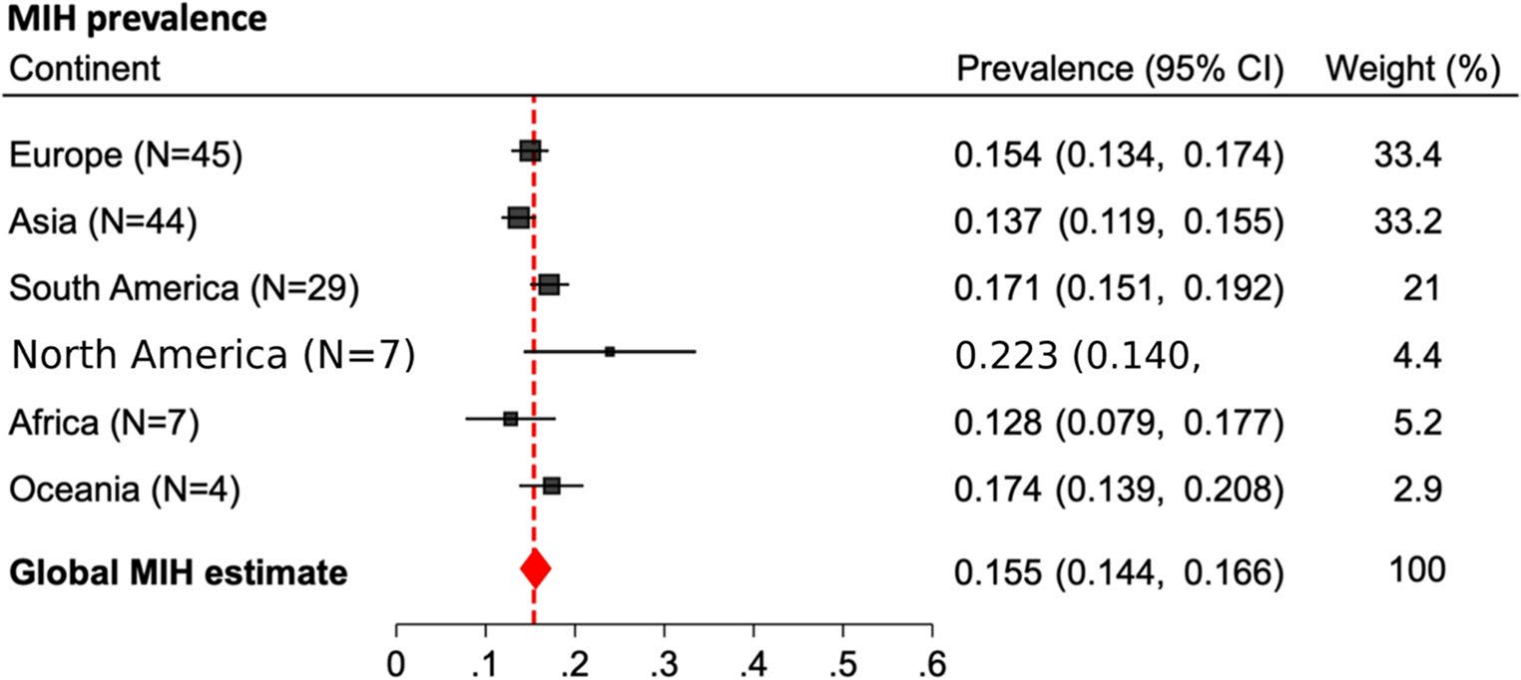
Corrected forest plot for the subgroup analyses of MIH prevalence per continent

The meta-analysis results have been updated in the attached table 1 and figure 1. The missing study presented data from a cohort of 337 children aged from 6 to 15 years old and influenced the calculations for North America (Table 1 and Figure 1). The meta-analysis now includes 139 studies and data from a total of 199,999 participants. For North America, the MIH prevalence estimate decreased slightly from 0.239 (95% CI: 0.144–0.334) to 0.223 (95% CI: 0.140–0.306). The forest plot for the presentation of global MIH prevalence was corrected accordingly (Figure). With an updated total number of seven studies from North America, it remains the continent with the highest reported MIH prevalence. Notably, the global prevalence estimate for MIH remained unchanged at 0.155 (95% CI: 0.144–0.166).

## Conclusion

Consequently, the addition of the overlooked study influenced slightly the MIH outcome for North America but had no impact on the previously drawn conclusions.

**Table 1:** Corrected meta-analysis results for the MIH prevalence in North America

Continent	Country	EH Prevalence	MIH Prevalence	M + IH Prevalence
North America (*N* = 7)	United States of America (*N* = 2)		0.109 (0.086–0.132)	0.051 (0.033–0.078)
	Mexico (*N* = 5)		0.268 (0.161–0.374)	
	**North America estimate**		**0.223 (0.140–0.306)**	**0.051 (0.033–0.078**)
Global estimate		**0.253 (0.200–0.306)**	**0.155 (0.144–0.166)**	**0.069 (0.060–0.077)**

## Data Availability

The dataset used in this study is available in the supplementary materials.
